# What drives people’s protective behaviors during the early stage of the COVID-19 pandemic in China

**DOI:** 10.3389/fpsyg.2022.781279

**Published:** 2022-10-21

**Authors:** Zhenjing Pang, Ce Zhao, Lan Xue

**Affiliations:** ^1^School of Public Policy and Management, Tsinghua University, Beijing, China; ^2^Beijing Language and Culture University, Beijing, China

**Keywords:** COVID-19, protective behavior, perceived threat, information seeking, information-processing strategy

## Abstract

This study systematically examined people’s protective behaviors against COVID-19 in China, and particular attention was given to people’s perceived threat and information-processing strategies. This study constructed a conceptual model and used structural equation modeling to explore this issue, and a questionnaire survey was conducted to collect data involving 4,605 participants during the early stage of the COVID-19 pandemic in China. The results showed that people’s initial information acquisition played an essential role in their behavioral responses; acquiring more initial information about COVID-19 would make them perceive a higher threat and present a higher demand for information, then making them more likely to seek and process information, and subsequently motivating their protective behaviors. In addition to increasing people’s information needs, the perceived threat could also strengthen the analytical assessment and affect protective behavior positively but failed to predict the experiential assessment. Driven by information need, information seeking significantly influenced protective behavior; it also facilitated analytical assessment and decreased experiential assessment, thus predicting people’s protective behaviors. Protective behaviors were spurred by analytical assessment but negatively influenced by the experiential assessment.

## Introduction

At the end of 2019, the novel coronavirus disease (COVID-19) outbreak began in Wuhan. This potentially fatal infectious disease was characterized by a steady speed of spread and transmitted from human to human through respiratory droplets or direct contact (Ranjit et al., 2021). The World Health Organization (WHO) classified it as a pandemic on 11 March 2020. In the past 2 years, the COVID-19 pandemic has affected most countries worldwide and caused a heavy loss of both life and economy. The Chinese government took many measures to curb the spread of COVID-19 and achieved staged success in the fight against this disease. There was no doubt that public compliance with practical health proposals was crucial in achieving this success.

The COVID-19 pandemic not only affected daily life and the economy but also shaped people’s behavior. Generally speaking, a significant crisis event reflects people’s historical experience and new characteristics in dealing with risk situations. Mainly, due to the lockdown policy, people live in a virtual environment built by information media, and risk information profoundly shapes the dimensions of people’s protective behaviors. Some people took positive action based on best practice guidelines, and some people failed to engage in protective behaviors. The variation in citizen response suggests that it is timely to explore the formation mechanism of people’s protective behaviors in the COVID-19 pandemic.

The unpredictable outbreak of COVID-19 has motivated studies of disease protective behaviors last year. For example, based on comparative analysis, a survey conducted by Ye et al. (2020) compared the adoption of basic, advanced, and excessive preventive behaviors in different groups of demographic characteristics. Liu and Mesch (2020) investigated factors related to the adoption of social distancing behaviors in China and Israel from the perspective of cultural differences. Chen and Chen (2020) compared prevention behaviors between urban and rural residents in China. Meier et al. (2020) compared public belief in the effectiveness of protective measures in the Netherlands, Germany, and Italy. Meanwhile, based on the theoretical foundation of cognitive behavior, some scholars explored the influencing factors associated with adopting preventive behaviors. For example, Storopoli et al. (2020) examined how personal cognition shaped prevention behaviors by applying the recreancy theory. Taking the protection motivation theory as the basic framework, Barati et al. (2020) tested the relationship between threat perception, coping appraisal, and prevention behavior.

Although previous studies have made substantial contributions to the protective behaviors against COVID-19, most of the conclusions were based on a single theoretical framework and comparative analysis. Most importantly, the information-processing strategies influencing the protective behaviors were still not clearly defined. Thus, a study exploring the formation mechanism of people’s protective behaviors is needed. This is especially true in China, where the COVID-19 outbreak began and aroused widespread concern. To address these issues, we constructed a conceptual model to better explain people’s protective behaviors and help public sectors improve behavior through the policy effectiveness of behavior guidance.

## Theory and hypotheses

The protective action decision model (PADM) proposed by is an essential framework for explaining people’s protective action decisions in response to imminent disasters or long-term hazard adjustments. The PADM emphasizes that people exposed to a potential risk receive risk information from outside and that the resulting risk perception is derived from the combination of that information. It also brings attention to people’s behavioral reactions intended to remove uncertainty about the risk and take appropriate protective actions. In the PADM, protective action decision-making begins with environmental cues, social cues, and warnings. This information initiates a series of pre-decisional processes that, in turn, elicit core perceptions of the ecological threat, alternative protective actions, and relevant stakeholders. These perceptions provide the basis for protective action decision-making, the outcome of which combines with situational facilitators and impediments to produce a behavioral response. The response can be information search, protective response (problem-focused coping), or emotion-focused coping. As the research stream evolved, a more recent version of PADM takes account of some other factors and integrates information flow into the model (Lindell and Perry, 2012). The new updated PADM indicated that some people who receive a warning might find that the available information is insufficient to justify a protective action positively. When they think time is available, people cope with the lack of knowledge by searching for additional information, and people commonly need additional information about the threat’s certainty, severity, and immediacy. The information search process begins with an information needs assessment arising from an individual’s judgment that the available information is insufficient to justify proceeding further in the protective action decision process. The PADM provides a systematic and comprehensive idea for understanding people’s protective behaviors under the risk situation of COVID-19. However, the PADM does not characterize information-processing strategies in detail. This is of particular importance to understanding protective behaviors because COVID-19 is a new risk situation and is not yet fully understood by people. Thus, the information-processing strategies of protective behaviors should be explained clearly.

In attempting to evaluate information to arrive at a judgment, the heuristic–systematic information-processing model (HSM) presents a careful understanding of these issues. According to the HSM, the strategy that people select to process information includes a dual-process model of systematic processing and heuristic processing, and this strategy makes a big difference in what individuals take away from these messages about risk and might affect their risk judgment. Systematic processing occurs when individuals make a judgment by carefully examining, comparing, and relating arguments; individuals usually require the information quality to meet higher standards before making a decision. On the contrary, heuristic processing occurs when individuals use simple decision rules to help them arrive at a judgment about the validity of a message. Individuals may spend less effort and fewer resources and often easily accept the information they hold or acquire from outside without questioning. Similarly, Slovic and Peters (2006) indicated that individuals have two modes of risk information assessment: analytical assessment and experiential assessment. The analytical assessment concludes with information integration and logical analysis, while the experiential assessment uses simple rules to arrive at a judgment (Slovic and Peters, 2006).

Two modes of information-processing work simultaneously or individually, and information sufficiency determines the two different processing modes. People are more motivated to use systematic processing or analytical assessment to choose subsequent behaviors if they have sufficient professional information. In contrast, limited information is an antecedent of heuristic processing or experiential assessment. Recently, scholars continually perfected information processing by integrating various behavioral theories and models into the original model. The risk information-seeking model (RISM) proposed by Griffin et al. (1999) further explained the phenomenon of purposeful seeking for specific information to make correct behavior decisions. Wei et al. (2016) and Zhu et al. (2016) integrated the RISM into the HSM and assumed information seeking and information need are the starting point and the internal driving force for information processing.

In addition, the mindsponge information-processing framework (MIPF for short) is also helpful for exploring the formation mechanism of protective behavior against COVID-19. The MIPF proposed by Vuong explains how a person receives and evaluates (filters) the information, accepts or rejects values, and updates related beliefs and behaviors in the process. Mindsponge is not only a coping mechanism aiming to solve internal conflicts but also a more inclusive model of cognition and behavior shifting process. The MIPF assumed that every person has a mindset consisting of a set of core cultural values or beliefs, which defines the person’s identity, perceptions, and behaviors (Vuong and Napier, 2015). The mindset is surrounded by a comfort zone driven by a multi-filtering information process detecting and connecting information. When information from the external environment enters the comfort zone, here the information is evaluated by the filtering system, information availability/accessibility and subjective cost–benefit judgments are the two fundamental conditions for a new piece of information to be accepted into the mindset (Nguyen et al., 2021). If both the objective availability and perceived accessibility of the information are guaranteed (the information needs to exist, be reachable, and be considered reachable to be received by the mind), it has to go through the cost–benefit judgments based on references of existing trusted values from the mindset including both rational and emotional-through many layers (Vuong et al., 2022a). The mindset absorbs and ejects information for the purpose of maximizing total perceived benefit and reducing total perceived cost for an individual. Information accepted into the mindset is integrated into one’s belief system and will affect subsequent decisions. If the information is accepted, it can move into the mindset and become a new trusted value. If the accepted information directly corresponds to a behavior (whether mental or physical), then that behavior will be carried out (Nguyen et al., 2021; Vuong et al., 2022a).

The mindsponge information-processing framework (MIPF) provides us with comprehensive insight into the protective behavior under the COVID-19. According to the MIPF, if a person is accessible to COVID-19 information, they may perceive risk caused by the virus (perceived threat or cost). When the information about the COVID-19 acquired by people is absorbed into the mindset, the value judgment and relevant behavior principles contained in the information about the epidemic will become the updated core beliefs in the mindset; then, it will influence the subsequent information processes and behaviors. For example, it may increase people’s demand for information and encourage people to seek more useful information related to COVID-19; it could also make people more cautious about the information and more rational in analyzing the information and then carrying out positive protective behaviors relatively (Vuong, 2022).

**Figure 1:**
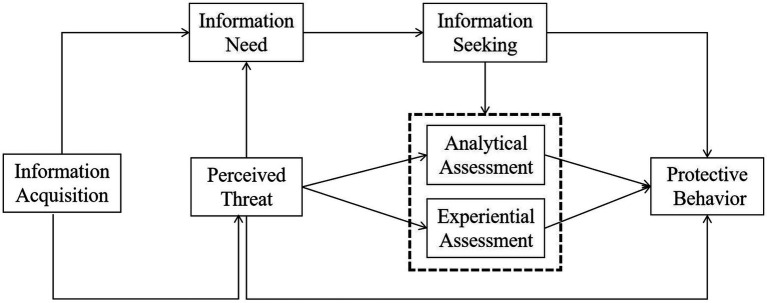
Conceptual model.

After the model combination and integration, we constructed the conceptual model (shown in Figure 1). This model adapts and synthesizes components from the PADM, HSM, RISM, and MIPF. Most variables were directly chosen from the applied models or replaced with relevant variables to fit the COVID-19 situation. The model assumes that people’s information acquisition and perception of risk simultaneously trigger information need and information seeking. Subsequently, two information-processing mechanisms are stimulated. Finally, people produce protective behaviors. The proposed hypotheses are presented in Table 1 and discussed in more detail as follows.

**Figure 2:**
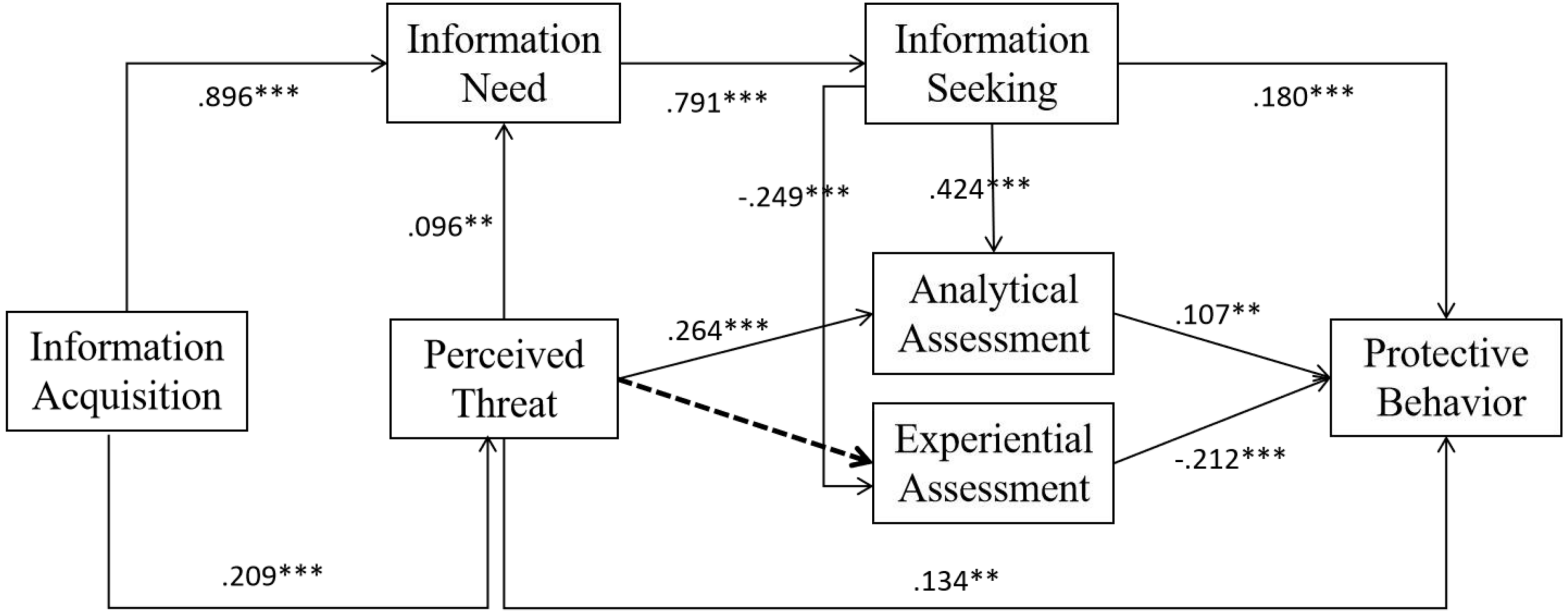
Results of conceptual model. ** *p <* 0 0.05 and *** *p <* 0.001.

**Table 1:** Developed hypothesis and causal relationships.

Hypothesis	Causal relationships			Developed hypothesis
H1	PT	←	IA	People who acquired more information about COVID-19 perceived more threat
H2	IN	←	IA	People who acquired more information about COVID-19 have stronger information need
H3	IN	←	PT	People who perceived more threat about COVID-19 tend to present higher information need
H4	IS	←	IN	People who exhibit higher information need about COVID-19 tend to exhibit higher information seeking
H5	AA	←	IS	People who seek more information about COVID-19 are more likely to process information using analytical assessment
H6	EA	←	IS	People who seek less information about COVID-19 are more likely to process information using experiential assessment
H7	AA	←	PT	People who perceived higher threat about COVID-19 are more likely to process information using analytical assessment
H8	EA	←	PT	People who perceived lower threat about COVID-19 are more likely to process information using experiential assessment
H9	PB	←	IS	People who seek more information about COVID-19 are more likely to take protective behavior
H10	PB	←	AA	People who process information using analytical assessment are more likely to take protective behavior
H11	PB	←	EA	People who process information using experiential assessment are less likely to take protective behavior
H12	PB	←	PT	People who perceived higher threat about COVID-19 are more likely to take protective behavior

### Information acquisition and Perceived threat

According to the PADM, some environmental cues, social cues, and socially transmitted warnings that people acquire are the initial factors of the information-processing chain associated with protective behaviors (Lindell and Perry, 2012). The transmission of risk information is based upon a six-component communication model of “source-channel-message-receiver-effect-feedback”. In the COVID-19 crisis, people acquired a certain amount of risk information through public sectors, traditional media, new media, and interpersonal channels. The data were generally fragmentary; the accuracy of the message may be less than desired.

In the PADM, risk perception is a central factor influencing people’s responses to threatening events (Lindell and Perry, 2012). Here, we developed a similar concept, “perceived threat” as an essential predictor of individuals’ behavioral reactions to adjusting to COVID-19; perceived threat denotes people’s initial perceptions about the threats caused by the adverse physical and social impacts. Many researchers have proved that information acquisition was a predictor of perceived threat (Brenkert-Smith et al., 2013; Wachinger et al., 2013), emphasizing perceived threat refers to people’s expectations of the personal impacts of a risk situation (Slovic and Peters, 2006), including risk-consequence perception, risk-probability perception, and risk-proximity perception (Lindell and Hwang, 2010; Yue et al., 2011). Perceived threat is not only a relatively subjective concept but is also highly correlated with uncertainty about the expected results. When people are exposed to certain risk information for a long time, individuals’ expectations related to the likelihood of personal physical and social impacts may be surrounded by an extensive and long-term perception of risks. These expected impacts include death, injury, property damage, and disruption to daily activities such as work, school, and shopping. Thus, we develop the following hypotheses:

*H1*: people who acquired more information about COVID-19 perceived more threat

### Information need and information seeking

Information need (IN) refers to the perceived gap between the sufficiency threshold of information required for specific goals and the amount of currently held information (Huurne and Gutteling, 2008). As the result of professional barriers, information asymmetry, and cognitive constraints, the information gap makes it difficult for people to evaluate COVID-19. People may need more information about the progress of the crisis to help assess risk. As they acquire more and more knowledge, people may hope that society can provide more up-to-date information through official or other channels. So people usually try to fill this gap by getting more information through different information sources because people who feel threatened often want to accurately assess the threat with information at a higher quality level (Lindell and Prater, 2010). Sufficient information can reduce the cognitive cost of using information and increase the benefit of obtaining it. Hence, the following hypotheses are proposed:

*H2*: people who acquired more information about COVID-19 have stronger information need

Meanwhile, the perceived threat is essential for predicting individual behavioral responses. People’s initial threat perception could lead to an increased feeling of uncertainty. This may make people aware that their information is insufficient in quantity and quality and motivate people to seek additional information to clearly understand the threat (Yue et al., 2011). Most previous studies confirmed threat perception was a driving force for information needs (Prati and Zani, 2013; Wang et al., 2016; Iwona et al., 2019). In the risk situation of COVID-19, people’s threat perception generally increases the feeling of fear and anxiety. They may desire to obtain more available information to justify an appropriate protective action, thus motivating them to seek additional information and logistical support for other protective behaviors. So the following hypotheses are proposed:

*H3*: people who perceived more threat about COVID-19 tend to present higher information need

Furthermore, many studies have explained the relationship between information need and information seeking. Just as the updated PADM mentioned, people who encounter a risk might find that the available information is insufficient to assess the risk and justify an appropriate protective behavior. This information gap is viewed as the key motivator for information seeking (Lindell and Perry, 2012). If people realize the available information was insufficient to assess risks, they may search for additional information about the threat’s certainty, severity, and immediacy. In the HSM, information need is also the motive force of information search (Wei et al., 2016). Griffin et al. (2008) used information insufficiency to describe the gap between individuals’ information held and their information needed and defined information seeking as the efforts of individuals to gather information. Many studies supported information need positively affects information seeking (Griffin et al., 1999; Moore, 2002; Huurne and Gutteling, 2008; Dunwoody and Griffin, 2014). In the COVID-19 crisis, people need more information about the epidemic’s progress due to the stay-at-home policy, hoping to acquire more timely information through various channels. Therefore, the following hypotheses are developed:

*H4*: people who exhibit higher information need about COVID-19 tend to exhibit higher information seeking

### Analytical assessment and experiential assessment

The HSM views information processing as an antecedent to attitude formation and behavior change and hypothesizes that people process information using the strategies of systematic processing and heuristic processing. Slovic and Peters (2006) used analytical assessment (effortful) and experiential assessment (superficial) instead of HSM to describe these information-processing strategies. Analytical assessment is the logical evaluation and comprehensive comparison with which individuals make judgments. On the contrary, the experiential assessment works when individuals use simple rules to help them arrive at a decision. There are many factors affecting people’s information-processing strategies. Information sufficiency is considered the essential determinant (Kahlor et al., 2010). When individuals carry sufficient information, they are more motivated and able to analyze the information related to this issue. On the contrary, insufficient information is a vital stimulant to the experiential assessment, and people who hold less information are more likely to process information rely on emotion and experience cues (Trumbo, 2010; Trumbo et al., 2010). However, this relationship has not been confirmed in the risk scenario of COVID-19; whether or not information seeking affects people’s information-processing strategies is yet to be determined. Therefore, the related hypotheses are developed:

*H5*: people who seek more information about COVID-19 are more likely to process information using analytical assessment

*H6*: people who seek less information about COVID-19 are more likely to process information using the experiential assessment

There are few studies on people’s risk perception influencing information-processing strategy. Hovick et al. (2011) examined the indirect effects of risk perception on systematic processing; the direct relationship is unclear in a particular risk situation. Wei et al. (2016) linked risk perception with systematic processing in the issue of the Volkswagen crisis and indicated risk perception is the key motivator for individuals to process information systematically, but in an inevitable public health crisis, the comprehensive relationships are still not defined clearly. When people are exposed to a risk, higher levels of threat perception motivate them to seek more information and increase their intentions to evaluate the risk further. This evaluation usually requires them to analyze the information with more effort. When people face the situation of COVID-19, the initially perceived threat increases the degree of the perceived threat and affects their intentions to adopt different information-processing strategies (Shadmi et al., 2020). Generally, people who have perceived more threats know more about COVID-19 and have an advantage in analytical thinking and logical reasoning when making protective behavior decisions. In comparison, people who perceive more minor threats are easier to draw a protective behavior decision through personal experience, emotion, and recommendations from others. Higher threat perception may inspire people’s analytical assessment, and the experiential assessment processing will be conserved. Therefore, the following hypotheses are developed:

*H7*: People who perceived higher threat about COVID-19 are more likely to process information using analytical assessment

*H8*: People who perceived lower threat about COVID-19 are more likely to process information using experiential assessment

## Protective behavior

Previous studies have already conducted an in-depth summary of people’s protective behaviors from the perspective of concept classification. Part of the findings reached a uniform conclusion, and some differed from others (Terpstra and Gutteling, 2008; Lindell and Perry, 2012). Many studies have identified that individuals who seek more information exhibited higher intentions to take protective behaviors for keeping themselves away from risk (Burton et al., 1993; Wei et al., 2016). In COVID-19, information asymmetry makes people search for more information and conduct an overall weighted evaluation of the severity of the epidemic, then motivating them to adopt protective behaviors to avoid risk positively. Therefore, the following hypothesis is developed:

*H9*: people who seek more information about COVID-19 are more likely to take protective behavior

However, few studies explain the impact of information-processing strategies on behavioral response. In particular, the effects of analytical assessment and experiential assessment on protective behavior have not been compared in a specific risk situation. Hovick et al. (2011) tested the relationship between systematic processing and protective behavior in a health crisis, and they concluded that people usually show positive health-protective actions with analytical assessment. In many other risk situations, information processing has also been identified in that individuals who process information with logical evaluation exhibit higher intentions to take actions to avoid the risk. In general, analytical assessment is driven by sufficient information and is conducted by analyzing and comparing, and then motivating people to take protective behaviors against COVID-19. The experiential assessment means an automatic processing strategy in which individuals respond to a stimulus without sufficient information and can be viewed as a lack of additional efforts and using experience, emotion, and following to evaluate, quickly leading to fewer sound judgments and negative protective behaviors when facing the COVID-19. Therefore, the following hypotheses are developed:

*H10*: people who process information using analytical assessment are more likely to take protective behavior

*H11*: people who process information using experiential assessment are less likely to take protective behavior

Perceived threats are believed to be crucial for people’s protective behavior. Most research on disasters has found that threat perception predicted warning responses, such as evacuation (Sorensen, 2000) and long-term risk adjustments (Lindell, 2013). These protective responses have been studied for hazards such as earthquakes, hurricanes, other coastal storms, floods, and volcanic eruptions (Dash and Gladwin, 2007). In this study, we plan to expand these studies in the context of COVID-19 and examine whether a perceived threat influences people’s protective behaviors. Therefore, the following hypothesis is developed:

*H12*: people who perceived higher threat about COVID-19 are more likely to take protective behavior

## Materials and methods

### Survey

To explore people’s protective behaviors against COVID-19, we conducted an online survey through Wenjuanxing, the most popular online survey platform in China. The questionnaire consisted of three parts: an introduction page, a variable page, and a socio-demographic characteristics page. After a brief introduction to thank respondents for their participation, some basic scenario information that introduced the progress and uncertainties of COVID-19 was presented. Then, a section of items was designed to identify scales of constructs. Finally, some questions investigating demographics were in the last section. The questionnaire was written in Chinese; although it was developed in English, we invited four bilingual risk researchers to help us translate it into Chinese and back-translate it into English. By comparing the different versions, we modified and deleted the contents that did not fit Chinese habits and culture to ensure the content validity of our questionnaire. Before the formal investigation, a pre-survey with a convenience sample of 110 students was conducted for further checking and refining the scenario information and measures. The duration of whole investigation process lasted from 15 February to 20 February 2020. A random sample of 5,780 respondents was interviewed online, 1,175 responses were invalid due to missing data, and the participants did not recognize two inverse questions embedded in the questionnaire; 4,605 valid questionnaires were used in this study.

Table 2 shows the summary statistics of socio-demographic characteristics of respondents, including gender, age, education, and registered residence. The gender ratio is almost equal, with 47.1% (*N* = 2,170) percent of the sample being male and 52.9% (*N* = 2,435) being female. As for the age distribution, the largest groups are between 21 and 30 (36.4%, *N* = 1,675) and 31 and 40 (40.9%, *N* = 1,685), followed by 6.2% (*N* = 285) of those respondents are under 20 years old, 13.2% (*N* = 610) are between 41% and 60%, and 3.2% (*N* = 150) are over 50 years old. In terms of education, the respondents are relatively well-educated, over half of the respondents (79.1%, *N* = 3,645) completed their college program, followed by a Master’s degree or above 13.9% (*N* = 640), and a small portion of the respondents (6.9%, *N* = 330) are high school or below. Finally, the registered residence falls into rural with 50.5% (*N* = 2,325) and urban with a percentage of 49.5% (*N* = 2,280).

**Table 2:** Demographic profile of respondents (*N* = 4,605).

Variables	Category	Frequency	Percentage (%)
Gender	Male	2,170	47.1
	Female	2,435	52.9
Age	20 or below	285	6.2
	21–30	1,675	36.4
	31–40	1,685	40.9
	41–50	610	13.2
	50–60	135	2.9
	61 or above	15	0.3
Education	Primary school and below	25	0.5
	junior middle school	95	2.1
	High school	200	4.3
	University(College)	3,645	79.1
	Master degree or above	640	13.9
Registered residence	Rural	2,325	50.5
	urban	2,280	49.5

### Measures

The measurement scale used in this study contained seven constructs, each of the variables was measured with multiple items based on previous literature and modified to fit the context of COVID-19 with a seven-point Likert scale.

In this paper, information acquisition is the initial amount of information related to COVID-19 people acquired from multiple channels. We measured people’s information acquisition using five items modified from the work of Pang (2020). People were asked how often they have heard about COVID-19 from the government, experts, family or friends, and traditional media (TV, newspaper, and radio), and how much information they have received from traditional media (TV, newspaper, and radio) and new media. In addition, the scores of all items varied from “never” to “very often” with a 7-point Likert scale.

Information need was measured by three items using a subjectively selected subset of items modified from Huurne and Gutteling (2008), and the measurement included “knowing more information about COVID-19 is necessary,” “I want more information related to COVID-19,” and “I hope to obtain a comprehensive understanding of COVID-19 through multiple channels.” The measurement of information seeking was also based on previous research conducted by Huurne and Gutteling (2008). The items mainly reflected the following three aspects: “It is necessary to search for information related to COVID-19,” “I am very pleased to search for information about COVID-19,” and “I search for comprehensive information about COVID-19 through multiple channels.” Perceived threat is the most critical construct to examine how people understand the risks of COVID-19, and people are more likely to focus on the perceived degree of consequence, probability, and proximity (Lindell and Hwang, 2010; Yue et al., 2011). Thus, in this study, we measured perceived threat by three items modified from Ranjit et al. (2017). The measurement was described as follows: “I am susceptible to getting COVID-19,” “I think COVID-19 poses a serious threat to my health,” and “I feel the virus is very close to me.” People could answer on a 7-point Likert scale ranging from “totally disagree” to “totally agree.”

The analytical assessment was measured through three items based on Slovic and Peters’s definition, and the items were listed as follows: “I learned about COVID-19 through a comparison of relevant information,” “I learned about COVID-19 by thinking about the most scientific information,” and “I tried to link this information with my major and interests.” As for measuring the experiential assessment, we also used Slovic and Peters’s (2006) concept as needed for our context, and the items were shown as follows: “I exerted little effort in learning about COVID-19,” “I formulated my judgments on COVID-19 by following the comments of others,” and “I made a risk evaluation on COVID-19 according to the intuition.” All items were measured on a seven-point Likert scale from “totally disagree” to “totally agree.”

The measurement of protective behavior is based on Lindell and Perry’s (2012) initial definition of coping behavior and adapted them to certain risk situations COVID-19. These items were measured as follows: “I wear masks and goggles when going out,” “I store enough protective equipment (e.g., masks, alcohol, food),” “I reduce contact with others,” “I spread scientific epidemic prevention knowledge to others,” “I put forward suggestions to the government for epidemic prevention,” and “I donate prevention equipment to the epidemic areas.” All measures were completed on seven-point Likert scales, where 1 indicated strong disagreement and 7 told strong agreement.

### Data analysis

Before empirically testing the measurement and structural models, our constructs’ descriptive statistics and correlations are presented in Table 3, including means (the means of the items), standard deviation, and correlation. The results reveal that people also expressed a strong need to search for more information (*M* = 5.86). Meanwhile, compared with empirical assessment (*M* = 4.97), people had a relatively high dependence on analytical assessment (*M* = 5.74) and placed a high value on the threat of COVID-19 (*M* = 4.98), and finally had a relatively high degree of protective behavior when facing the epidemic (*M* = 5.25). For the correlations between various constructs, the correlation analysis results verify the relationship assumed by the conceptual model, and it is appropriate to conduct further analysis.

**Table 3:** Means, standard deviation, and correlation.

M	5.15	6.00	5.86	4.98	4.97	5.74	5.25
SD	1.03	1.05	1.11	1.27	0.91	0.95	0.86
Correlation	IA	IN	IS	PT	EA	AA	PB
IA	1						
IN	0.487^***^	1					
IS	0.546^***^	0.791^***^	1				
PT	0.148^***^	0.191^***^	0.158^***^	1			
EA	−0.355^***^	−0.369^***^	−0.373^***^	−0.179^***^	1		
AA	0.415^***^	0.510^***^	0.470^***^	0.185^***^	−0.379^***^	1	
PB	0.192^***^	0.299^***^	0.270^***^	0.086^**^	−0.215^***^	0.219^***^	1

* *p* < 0.1;

** *p* < 0.05;

*** *p* < 0.001.

According to known procedures, the data analysis consists of two stages. First, a measurement model was created and estimated by confirmatory factor analysis (CFA) to determine whether the questionnaire items measured their intended constructs correctly, namely the reliability and validity tests. In the second stage, when measurement quality was confirmed, a structural model was established and conducted with SEM analysis to verify the hypothesized relationships of the proposed model under the condition of a satisfactory measurement model. Confirmatory factor analysis (CFA) was implemented to evaluate the adequacy of the measurement model.

As shown in Table 4, the reliability and validity results showed that the composite reliability values were over the threshold value of 0.70. Cronbach’s coefficients were over the threshold value of 0.70 significantly. The CITCs of all items satisfied the general recommended level of 0.70. Standardized loading was greater than 0.7, and the value of *p* was significantly related to its latent construct (*p* < 0.001). All AVEs were more than 0.5, and the square root of AVEs was greater than the cross-correlations between constructs. Thus, we can conclude that the measurement model had adequate reliability and validity. As shown in Table 5, the goodness-of-fit measures for the overall confirmatory model indicated that the chi-square ratio, REMSEN, GFI, CFI, PGFI, PNFI, PCFI, AGFI, TLI, and NFI were also over the threshold. Thus, the findings indicate that the conceptual model satisfactorily fits the data.

**Table 4:** Confirmatory factor analysis results for measurement model.

Constructs	Labels	Loadings	CITC	CR	Cronbach’s *α*	AVE
Information acquisition	IA1	0.782^***^	0.789	0.924	0.819	0.945
	IA2	0.841^***^	0.764			
	IA3	0.868^***^	0.751			
	IA4	0.811^***^	0.795			
	IA5	0.835^***^	0.819			
Information need	IN1	0.948^***^	0.918	0.933	0.942	0.956
	IN2	0.971^***^	0.876			
	IN3	0.924^***^	0.952			
Information seeking	IS1	0.945^***^	0.907	0.917	0.936	0.936
	IS2	0.967^***^	0.868			
	IS3	0.918^***^	0.947			
Perceived threat	PT1	0.849^***^	0.813	0.906	0.845	0.916
	PT2	0.867^***^	0.836			
	PT3	0.814^***^	0.786			
Analytical assessment	AA1	0.839^***^	0.813	0.914	0.838	0.929
	AA2	0.878^***^	0.837			
	AA3	0.867^***^	0.822			
Experiential assessment	EA1	0.891^***^	0.852	0.937	0.843	0.941
	EA2	0.890^***^	0.858			
	EA3	0.876^***^	0.843			
Protective behavior	PB1	0.801^***^	0.827	0.922	0.860	0.932
	PB2	0.898^***^	0.858			
	PB3	0.914^***^	0.885			
	PB4	0.876^***^	0.845			
	PB5	0.880^***^	0.834			
	PB6	0.871^***^	0.893			

* *p <* 0.1;

** *p <* 0.05;

*** *p <* 0.001.

**Table 5:** Goodness-of-fit statistics for structural model.

Index		Threshold	Acceptance
*x^2^/df*	3.335	<5.0	Passed
RMSEA	0.051	<0.08	Passed
GFI	0.903	>0.9	Passed
PGFI	0.502	>0.5	Passed
AGFI	0.906	>0.9	Passed
TLI	0.908	>0.9	Passed
CFI	0.919	>0.9	Passed
NFI	0.908	>0.9	Passed
PNFI	0.513	>0.5	Passed
PCFI	0.524	>0.5	Passed

## Results

As shown in the structural model results in Table 6 and Figure 2, the result revealed that the model’s performance effectively supported the conceptual model; all but two paths (H8) achieved statistical significance at the level of 0.1 or better.

**Table 6:** Results of structural equation modeling.

Hypothesis	Causal relationships			Estimate	SE	*P*	Supported (YES/NO)
H1	PT	←	IA	0.209	0.041	<0.001^***^	YES
H2	IN	←	IA	0.896	0.082	<0.001^***^	YES
H3	IN	←	PT	0.096	0.047	0.021^**^	YES
H4	IS	←	IN	0.791	0.024	<0.001^***^	YES
H5	AA	←	IS	0.424	0.032	<0.001^***^	YES
H6	EA	←	IS	−0.249	0.027	<0.001^***^	YES
H7	AA	←	PT	0.264	0.040	<0.001^***^	YES
H8	EA	←	PT	−0.020	0.020	0.309	NO
H9	PB	←	IS	0.180	0.040	<0.001^***^	YES
H10	PB	←	AA	0.107	0.051	<0.001^**^	YES
H11	PB	←	EA	−0.212	0.057	<0.001^***^	YES
H12	PB	←	PT	0.134	0.044	0.005^**^	YES

* *p <* 0.1;

** *p <* 0 0.05;

*** *p <* 0.001.

The overall model showed that the path from information acquisition to the perceived threat was statistically significant (β = 0.209, *p* < 0.001), and this result indicated confirmatory evidence for H1. In the case of the relationship between information acquisition, information need, and information seeking, the empirical results showed that the influencing path from information acquisition to information need (β = 0.896, *p* < 0.001) and the path from the information need to information seeking (β = 0.791, *p* < 0.001), just as expected, were positive signs, and both H2 and H4 were confirmed statistically.

As for the impact of perceived threat on information need, statistics suggested a significant influence path (β = 0.096, *p* < 0.05), and H3 was supported as predicted. Concerning the impact of information seeking on information-processing strategy in the formation of protective behavior toward COVID-19, the paths from information seeking to analytical assessment (β = 0.424, *p* < 0.001) and experiential assessment (β = −0.249, *p* < 0.001) were predicted to be positive and negative, respectively, and the results confirmed the authenticity of H5–H6. The predictors of information processing showed that perceived threat also played an important role. The result showed that perceived threat had significantly positive influences on analytical assessment (β = 0.264, *p* < 0.001), whereas failing to predict the experiential assessment (β = −0.020, *p* = 0.309) substantially, so the hypothesis H7 was supported in the model, while H8 was not confirmed.

Finally, we found that information seeking was positively related to people’s protective behaviors, and H9 was supported with a significant coefficient (β = 0.180, *p* < 0.001). Furthermore, the analytical assessment had significantly positive influences on protective behaviors (β = 0.107, *p* < 0.05), while the experiential assessment showed hostile relations (β = −0.212, *p* < 0.001), perceived threat appeared to have significantly positive influences on people’s protective behavior (β = 0.134, *p* < 0.05), and H10–H12 were supported as the conceptual model expected.

## Discussion

The PADM, HSM, RISM, and MIPF have unique advantages in explaining people’s protective behaviors and laid a stable theoretical foundation for exploring the formation mechanism of protective behavior against COVID-19. Based on the above theories and models, this study constructed a conceptual model and systematically examined the formation mechanism of people’s protective behaviors in the COVID-19 pandemic, and particular attention was given to perceived threat and information processing. The main findings and innovative insights were discussed in the following.

Unlike many previous studies that pay attention to subjective or objective knowledge (Yechiel et al., 2009; Soffer et al., 2010; Fitch-Martin et al., 2018), this study examined the initial information acquisition played in people’s protective behaviors, which many scholars have ignored. Although this study was the first to explore the influence of information acquisition on the formation of defensive behaviors in the public health crisis of COVID-19, the findings concluded that information acquisition was a critical predicting factor of information need, and people with more information acquisition about COVID-19 increased their desire for more information.

COVID-19 was a fatal infectious disease; people usually cared about the influence on their daily lives, the chance of being infected, and how far the threat was from oneself. Our findings confirmed that people with more information acquisition could perceive a higher threat level associated with COVID-19. It should also be noted that people who perceived a higher threat of this epidemic were more likely to present a higher demand for information and then inspire their intentions to seek more information related to COVID-19 for further risk judgment. This finding revealed that information need was a significant predictor of information seeking because COVID-19 was considered extremely dangerous; people who lacked sufficient information to assess the overall risk of the epidemic preferred to obtain more information and then showed motivation to seek additional information. These results were consistent with previous studies (Huurne and Gutteling, 2008; Wei et al., 2017).

In addition, this study divided information-processing strategies into two strategies: analytical assessment and experiential assessment. The analysis results showed that information seeking was significant in predicting analytical assessment and experiential assessment of risk information associated with COVID-19. When people feel that they have sufficient information through information seeking, they prefer to process information with a systematical way of logical reasoning, rather than simply processing in which people respond to COVID-19 without additional efforts to evaluate the information. Furthermore, the perceived threat was also crucial in predicting people’s decision strategies as a psychological factor. Unlike some previous studies that only examined the impact of perceived threats on relevant analytical assessment or systematic processing in various risk situations (Hovick et al., 2011; Wei et al., 2016), the relationships between perceived threat and these two strategies were first tested in this study. The conclusion indicated that people’s threat perception was the strengthening determinant of analytical assessment. In contrast, perceived threat failed to predict the experiential assessment significantly, confirming that those who thought COVID-19 was dangerous took more effort and usually used logical evaluation and comprehensive comparison to process information regarding COVID-19.

Finally, this study focused on people’s protective behaviors during the COVID-19 crisis. According to the estimated results, perceived threat and information seeking were two critical factors predicting people’s protective behaviors, whose effects acted in both direct and indirect paths. To be more specific, information seeking was proved to have a significantly positive influence on people’s protective behaviors, and people who obtained more information and had a high estimation level of threat of COVID-19 could be motivated to adopt protective behaviors. Meanwhile, as a psychological mechanism, information-processing strategy had a crucial mediating role in the relationship between information seeking, perceived threat, and protective behavior. On the contrary, analytical assessment and experiential assessment were both antecedents directly affecting people’s protective behaviors. Concretely speaking, protective behaviors were negatively influenced by the experiential assessment, but positively influenced by analytical assessment. A possible explanation was that analytical assessment could motivate people to approach the epidemic with caution, then take positive protective actions. In contrast, the experiential assessment increased casual judgment toward COVID-19, leading to non-stressful prevention behaviors. What is more, through the mediation mechanism of information processing, people who had a high threat perception and searched for more information could be inspired to adopt an analytical assessment to analyze existing information comprehensively and reduce intention to apply the experiential assessment, ultimately strengthening the coping behaviors for protecting themselves from the threat of COVID-19 indirectly.

## Conclusion

This study systematically examines people’s protective behaviors to the public health crisis of COVID-19 in China. Particular attention was given to people’s perceived threats and information-processing strategies influencing their protective behaviors. We constructed a conceptual model and used structural equation modeling to explore this issue. A questionnaire survey was conducted to collect data involving 4,605 participants in the first wave of the COVID-19 outbreak in China. The results showed that people’s initial information acquisition played an essential role in their behavioral responses to the crisis. Acquiring more initial information about COVID-19 would make them perceive a higher threat and present a higher demand for information, making them more likely to seek and process information and subsequently motivating their protective behaviors. In addition to increasing people’s information needs, the perceived threat could also strengthen the analytical assessment and positively affect protective behavior but failed to predict the experiential assessment. Driven by information need, information seeking had a significantly positive influence on protective behavior. It also facilitated analytical assessment and decreased experiential assessment, thus predicting people’s protective behaviors. Protective behaviors are spurred by analytical assessment but negatively influenced by the experiential assessment.

The main contribution of this study was enriching the current research on the issues of protective behaviors and providing new insights into the formation mechanism of protective behaviors in the public health crisis of COVID-19:

1. This study extended the application range of the protective action decision model, the heuristic–systematic information-processing model, the risk information-seeking model, and the mindsponge information-processing framework. Based on these existing theories and frameworks, we developed a new model for understanding the public’s protective behaviors from the perspective of information flow, which provided empirical validations to the PADM, HSM, RISM, and MIPF.
2. This study linked perceived threat with information-processing strategies creatively and empirically tested the effects of various predictor variables on protective behaviors in detail, especially the crucial role of information-processing strategies.
3. Our survey was conducted during the first wave of the COVID-19 outbreak, and the data were representative.

This study also has some practical implications. This article enlightened us: in public health crises like COVID-19, an information promotion strategy is crucial to social risk communication and protective behavior guidance. The government should establish diversified and institutionalized information disclosure mechanisms and proactively release all kinds of information on time through various channels. For example, the government affairs hotline, government official website, WeChat, Weibo, and other new media channels should be applied flexibly to fill the gap between the sufficiency threshold of information required and the amount of currently held information for understanding risk situations. Especially, sharing scientific knowledge with people by developing more open ways of public education is needed. The discourse system should return to the scientific field to make people cautious about the risk situation and urge them to analyze the information with systematic thinking to take positive protective behaviors. At the same time, we should also recognize various reasons for improper protective behavior. However, the conditions for negative protective behavior have a common feature: The middle area of behavioral decision-making lacks factual information, knowledge information, analytical ideas, emotional feelings expression, and evaluation framework that can be used, compared, shared, and selected. Therefore, information dissemination alone cannot fundamentally eliminate the conditions for improper protective behavior. In addition to the public sector, the guidance of protective behaviors includes different unofficial social figures, such as experts, scholars, professionals, media reporters, opinion leaders, front-line staff, and self-media bloggers. These social figures can become essential supplements to the public sector in terms of behavior guidance, take on some roles that the public sector is inconvenient or unable to undertake, and play some important roles that the public sector cannot effectively play. To effectively play the vital role of social people in guiding protective behaviors, we need to provide corresponding institutional guarantee conditions. On the one hand, opinion leaders should be encouraged to guide protective behavior by providing factual information, professional knowledge, rational analysis ability, experience sharing ability, feeling expression ability, and emotional evaluation ability. Appropriate institutional space is reserved, and there is no need to demand that their expressions be the same as those of the public sector.

This article also enlightened us: in an emerging pandemic like COVID-19, until a vaccine is available, non-pharmaceutical interventions (rather than non-medical) are the primary measures to control the outbreak. To date, to control the pandemic, different countries have explored different non-drug interventions. These measures can be summarized as travel restrictions, social distancing, and personal protection measures, including canceling large mass gatherings, closing educational institutions, border restrictions, increasing personal protective equipment, conducting risk communication, strengthening public awareness and education effectiveness, providing assistance to vulnerable groups, and psychological counseling for the public.

The limitations of this study should be acknowledged. First, the study only focuses on people’s protective behaviors in China, and differences may exist in different countries because of cultural differences. Future endeavors should perform some comparative studies. Second, there may be some other factors that are not being considered, such as subjective value and objective knowledge. Hence, the generalization of the results in this study may be constrained, and future studies should consider these matters. Moreover, social vaccination is a vital protective behavior against the COVID-19 (Vuong et al., 2022b), but it has been not mentioned throughout the study, because the current study’s data were collected prior to the production of COVID-19 vaccines, future studies should present a careful understanding of vaccination in the context of protective behavior, especially the formation mechanism of vaccination intention should be the focus of research.

## Data availability statement

The original contributions presented in the study are included in the article/supplementary material, further inquiries can be directed to the corresponding author.

## Author contributions

ZP and LX were the principal investigator. CZ and ZP collected and analyzed the data under the supervision of LX. ZP and LX designed the study and contributed to materials and analysis tools. ZP and CZ contributed to the writing of the manuscript. LX provided guidance to the manuscript. All authors contributed to the article and approved the submitted version.

## Funding

This work was supported by The National Social Science Fund of China (Grant no. 22CSH014); China Postdoctoral Science Foundation (Grant no. 2022T150363).

## Conflict of interest

The authors declare that the research was conducted in the absence of any commercial or financial relationships that could be construed as a potential conflict of interest.

## Publisher’s note

All claims expressed in this article are solely those of the authors and do not necessarily represent those of their affiliated organizations, or those of the publisher, the editors and the reviewers. Any product that may be evaluated in this article, or claim that may be made by its manufacturer, is not guaranteed or endorsed by the publisher.
